# Abnormal Static and Dynamic Local-Neural Activity in COPD and Its Relationship With Pulmonary Function and Cognitive Impairments

**DOI:** 10.3389/fnhum.2020.580238

**Published:** 2021-01-15

**Authors:** Zhi Lv, Qingqing Chen, Yinling Jiang, Panpan Hu, Lei Zhang, Tongjian Bai, Kai Wang, Yongsheng Wang, Xiaoyun Fan

**Affiliations:** ^1^Department of Geriatric Respiratory and Critical Care Medicine, The First Affiliated Hospital of Anhui Medical University, Hefei, China; ^2^Department of Pulmonary, The Second People's Hospital of Hefei (The Affiliated Hefei Hospital of Anhui Medical University), Hefei, China; ^3^The Fifth Ward, Department of Tuberculosis, Anhui Chest Hospital, Hefei, China; ^4^Department of Neurology, The First Affiliated Hospital of Anhui Medical University, Hefei, China

**Keywords:** chronic obstructive pulmonary disease, semantic memory, resting-state functional MRI, dynamic, amplitude of low-frequency fluctuation

## Abstract

Patients with chronic obstructive pulmonary disease (COPD) are characterized by attenuated pulmonary function and are frequently reported with cognitive impairments, especially memory impairments. The mechanism underlying the memory impairments still remains unclear. We applied resting-state functional magnetic resonance imaging (RS-fMRI) to compare the brain local activities with static and dynamic amplitude of low-frequency fluctuations (sALFF, dALFF) among patients with COPD (*n* = 32) and healthy controls (HC, *n* = 30). Compared with HC, COPD patients exhibited decreased sALFF in the right basal ganglia and increased dALFF in the bilateral parahippocampal/hippocampal gyrus. The reduced the left basal ganglia was associated with lower oxygen partial pressure. Besides, the increased dALFF in the left hippocampal/parahippocampal cortex was associated with poor semantic-memory performance and the increased dALFF in the left hippocampal/parahippocampal cortex was associated the forced vital capacity. The present study revealed the abnormal static and dynamic local-neural activities in the basal ganglia and parahippocampal/hippocampal cortex in COPD patient and its relationship with poor lung function and semantic-memory impairments.

## Introduction

Chronic obstructive pulmonary disease (COPD) is a chronic disease of the lungs and a leading cause of significant mortality and disability (Watz et al., 2008). According to the WHO, COPD is the fourth leading cause of death and will be the third leading cause of death by 2030. Cognitive dysfunction is one of the most important comorbidities of COPD, which must be cautiously considered. It has been reported that 12–88% of patients with COPD present with cognitive impairments (Hynninen et al., 2005), either globally or in single cognitive domains (Dodd et al., 2010), especially memory deficits (Cleutjens et al., 2014). However, the pathogenesis of cognitive impairments remains unclear.

Multiple studies have revealed the relationship between cerebral structural lesions and poor respiratory function (Liao et al., 1999; Sachdev et al., 2006). Considering the direct relation between hypoxia and neuronal functional activity, COPD patients may be more susceptible to neuronal functional alterations. Hence, it is more reasonable from a functional view to explore the neural substance underlying cognitive impairments in COPD. Indeed, it is widely known that cognitive processing requires activity in the relevant cerebral regions (task-related neuronal activity). However, compared with task-related neuronal activity, spontaneous neuronal activity actually consumes more brain energy (Raichle and Mintun, 2006) and has been linked with multiple cognitive processes, such as spatial orientation and memory (Greicius et al., 2003). Spontaneous neuronal activity refers to the activity intrinsically generated by the brain that is not attributable to particular information inputs or outputs (Fox and Raichle, 2007).

Recently, spontaneous activity has been indexed by various approaches, such as resting-state functional connectivity (RSFC) and regional homogeneity (ReHo). These indexes reflect functional coupling between distinct brain regions, spatially remote and local, respectively (Friston, 2002; Zang et al., 2004), but not direct neuronal activity. As regional measures of spontaneous activity, the regional amplitude of low-frequency fluctuations (ALFF) has been extensively used to represent spontaneous neuronal activity (Zang et al., 2007). The ALFF were shown to be closely associated with other classical measures of brain activation, such as PET (Tomasi et al., 2013), and have been widely used to measure the neural substrates of several cognitive processes, including language learning and working memory (Zou et al., 2013; Deng et al., 2016). Besides, abnormal ALFF have been linked to cognitive dysfunction in various neurological disorders, such as Alzheimer's disease (Wang et al., 2011). Recently, there have also been studies that investigated the alterations in neuronal activity in patients with COPD using ALFF (Wenjing et al., 2018; Lu et al., 2019). For example, Lu et al. (2019) found decreased ALFF in bilateral basal ganglia areas and the aberrant ALFF value was correlated with PaO_2_ and the pulmonary ventilation function.

However, most of these studies on local spontaneous neuronal activity have focused on measuring static ALFF (sALFF), which is based on the implicit assumption that brain activity remains temporally stationary during the entire scanning period. Recently, mounting evidence has suggested there are time-varying characteristics of brain local activity (Hutchison et al., 2013; Allen et al., 2014; Liu et al., 2017), which was always indexed by the dynamic amplitude of low-frequency fluctuation (dALFF). Importantly, the dALFF has been frequently associated with cognitive performance in healthy people (Fornito et al., 2012) and patients with cognitive impairments (Fiorenzato et al., 2019). However, whether patients with COPD exhibit abnormal dALFF remains unclear. In the present study, we aimed to explore the static and dynamic local neuronal activities and their relationships to cognitive deficit in patients with COPD. We assumed that COPD patients present aberrant static and dynamic ALFF and these abnormal local activities may be related to cognitive impairments in COPD patients.

## Materials and Methods

### Participants

Thirty-two patients with COPD were recruited from the Hefei Second People's Hospital, Hefei, China. The patients were diagnosed according to the Global Initiative for Chronic Obstructive Lung Disease (GOLD) guidelines from 2013 and met other necessary inclusion criteria: (1) no current mental disorders or neurological illness or related history; (2) no history of substance abuse; (3) no comorbidities such as diabetes, liver failure, cardiovascular disease, neurological disorders, or malignant tumor; (4) years of schooling >5; (5) eligible head motion (<3 mm, 3°); (6) no claustrophobia or other contraindications of an MRI scan. Finally, thirty-two patients were included in the final analysis. Thirty healthy subjects were also enrolled and matched according to gender, age, and education via local advertisements. All participants were carefully screened in a diagnostic interview to rule out current or past significant medical illness or mental disorders. The present study was approved by the Anhui Medical University Ethics Committee, and written informed consent was obtained from all participants.

### Laboratory Tests

Within 3 days of MRI scanning, a standardized pulmonary function test was performed to evaluate the function of pulmonary ventilation in all participants. In addition, an arterial blood gas analysis was performed for all patients.

### Cognitive Test

The present study primarily focused on the semantic memory, assessed by a category verbal fluency test (CVFT). During the CVFT, participants were required to say as many words as possible describing a vegetable within 1 min. One point was scored when participants gave a correct term for the correct description of the vegetable. The total score was used as a record of memory performance for further analysis.

### MRI Data Acquisition

Structural and functional MRI images of participants were acquired at the University of Science and Technology of China, Anhui Province, with a 3-T scanner (Discovery GE750w, General Electric). Before image acquisition, all participants were asked to keep their eyes closed and body still, and not to think of anything in particular. T1-weighted anatomical images were acquired in the sagittal orientation [TR/TE = 8.16/3.18 ms; flip angle = 12°; field of view (FOV) = 256 × 256 mm^2^; voxel size = 1 × 1 × 1 mm^3^; slice thickness= 1 mm; 188 slices]. Functional MRI (BOLD) images were composed of 217 echo-planar imaging volumes (TR/TE = 2,400/30 ms; flip angle = 90°; FOV = 192 × 192 mm^2^; voxel size = 3 × 3 × 3 mm^3^; slice thickness = 3 mm; matrix size = 64 × 64; 46 continuous slices).

### Functional Data Preprocessing

Functional MRI data were preprocessed using the Data Processing Assistant for Resting-State Functional MR Imaging toolkit (Chao-Gan and Yu-Feng, 2010). For each participant, we applied the following processing steps: discarding the first 10 volumes to allow for magnetization equilibrium; slice timing correction; realignment to account for head motion; co-registered structural images with the mean functional image and then segmented into gray matter, white matter, and cerebrospinal fluid; the gray matter maps were non-linearly co-registered to the tissue probability maps in the Montreal Neurological Institute (MNI) space; normalization functional volume to the MNI space using the parameters estimated during non-linear co-registration at a resolution of 3 × 3 × 3 mm^3^; nuisance regressors with 24 Friston motion parameters, white matter high signal, cerebrospinal fluid signal and global signals as regressors; filtering with a temporal band pass of 0.01–0.1 Hz, and spatial smoothing (Gaussian kernel = 4 × 4 × 4 mm^3^). Finally, motion scrubbing was conducted using the cubic spline method to minimize the influence of the time points with high motion [frame-wise displacement (FD) >0.5], as well as that of one time point prior to, and two time points following, each of these high motion time points.

### Static Amplitude of Low-Frequency Fluctuations (sALFF)

After preprocessing, the filtered time series was converted to the frequency domain using a fast Fourier transform. The square root of the power at each frequency was calculated to obtain amplitude values. The ALFF was calculated as the sum of the amplitude values in the 0.01–0.1 Hz low-frequency power range. To reduce the global effects of variability across participants, the ALFF was normalized to the mean within-brain ALFF value for each participant.

### Dynamic Amplitude of Low-Frequency Fluctuations (dALFF)

The sliding window approach was used to calculate the dALFF using DynamicBC software (Liao et al., 2014). Based on previous recommendations, we chose 30 repetition times (TRs) (72 s) as the window length (Leonardi and Van De Ville, 2015; Li et al., 2018), and the window was shifted by 40% of the window length (12 TRs, 28.8 s). There were 207 TRs for our data and, hence, 15 windows constituted the full time series. For the time series in each window, the ALFF map was calculated using the procedures described above for the sALFF calculation. To study the temporal variability of ALFF, the standard deviation (SD) of ALFF at each voxel was calculated across sliding-window dynamics, then the coefficient of variation (CV: SD/mean) map was calculated, i.e., dALFF. Additionally, selected parameters, especially the length of window and span (overlap), are key factors in the computation of dynamics. To validate our findings, we conducted auxiliary analyses with another span (one TR, 2.4 s) and two window lengths [40 TRs (96 s) and 50 TRs (120 s)].

### Statistical Analysis

The Pearson's χ^2^ test and two-sample *t*-tests were applied to compare the demographic and clinical characteristics of the two groups (gender, age, educational years, clinical symptoms, and semantic memory performance) with SPSS 23. Voxel-wise two-sample *t*-tests within the gray matter mask were performed to quantitatively compare the differences in the sALFF and dALFF values between the two groups with gender, age, educational years, and head motion indexed by frame-wise displacement (FD) as covariates with the software of DPABI (http://rfmri.org/dpabi). Statistical maps for sALFF and dALFF were corrected using the Gaussian Random Field method at the threshold of *p* < 0.001 at the voxel level and *p* < 0.05 at the cluster level (cluster size >40 voxel). Spearman's correlation analyses were conducted to examine the associations between the significantly different sALFF and dALFF values between groups and behavioral performance. Significance for correlations was determined by *p* < 0.05 (two-tailed), with no correction.

## Results

### Demographic and Clinical Characteristics

Thirty-two patients with COPD and 30 healthy controls were included in the final analysis. No significant differences were found between the two groups in terms of age or gender. Compared with the HC, participants with COPD had lower scores in the CVFT. Table 1 presents the demographic characteristics of the two groups.

**Table 1:** Demographic and clinical characteristic.

	**COPD**	**HC**	**^**b**^T value/**χ**^2^**	***p*-value**
Gender (Male/female)^a^	30/2	27/3	0.29	0.59
Age	71.94 ± 5.52	71.10 ± 3.95	0.68	0.50
Educational years	6.03 ± 2.60	6.17 ± 2.13	−0.22	0.82
PH	7.40 ± 0.040			
PaO_2_	83.68 ± 22.87			
PaCO_2_	43.75 ± 10.70			
FEV_1_	0.95 ± 0.44			
FVC	1.84 ± 0.71			
FEV_1_/FVC	0.51 ± 0.088			
CVFT	12.69 ± 2.38			
sALFF in BG	0.67 ± 0.10	0.80 ± 0.11	−4.97	<0.001
dALFF in LHIP	0.29 ± 0.08	0.19 ± 0.04	6.03	<0.001
dALFF in RHIP	0.25 ± 0.05	0.18 ± 0.04	6.76	<0.001

a *Data are presented as mean ± standard deviation except Gender*.

b *Comparisons were performed using the chi-square test for the variable of Gender and independent samples t-tests for other variables*.

*COPD, chronic obstructive pulmonary disease; HC, healthy control; PH, pondus hydrogenii; PaO_2_, partial pressure of oxygen; PaCO_2_, partial pressure of carbon dioxide; FVC, forced vital capacity; FEV_1_, forced expiratory volume in the first second; CVFT, category verbal fluency test; sALFF, static amplitude of low-frequency fluctuations; dALFF, dynamic amplitude of low-frequency fluctuations; BG, basal ganglia; RHIP, right hippocampal/parahippocampal cortex; LHIP, hippocampal/parahippocampal cortex*.

### Group Comparison of sALFF

**Figure 1:**
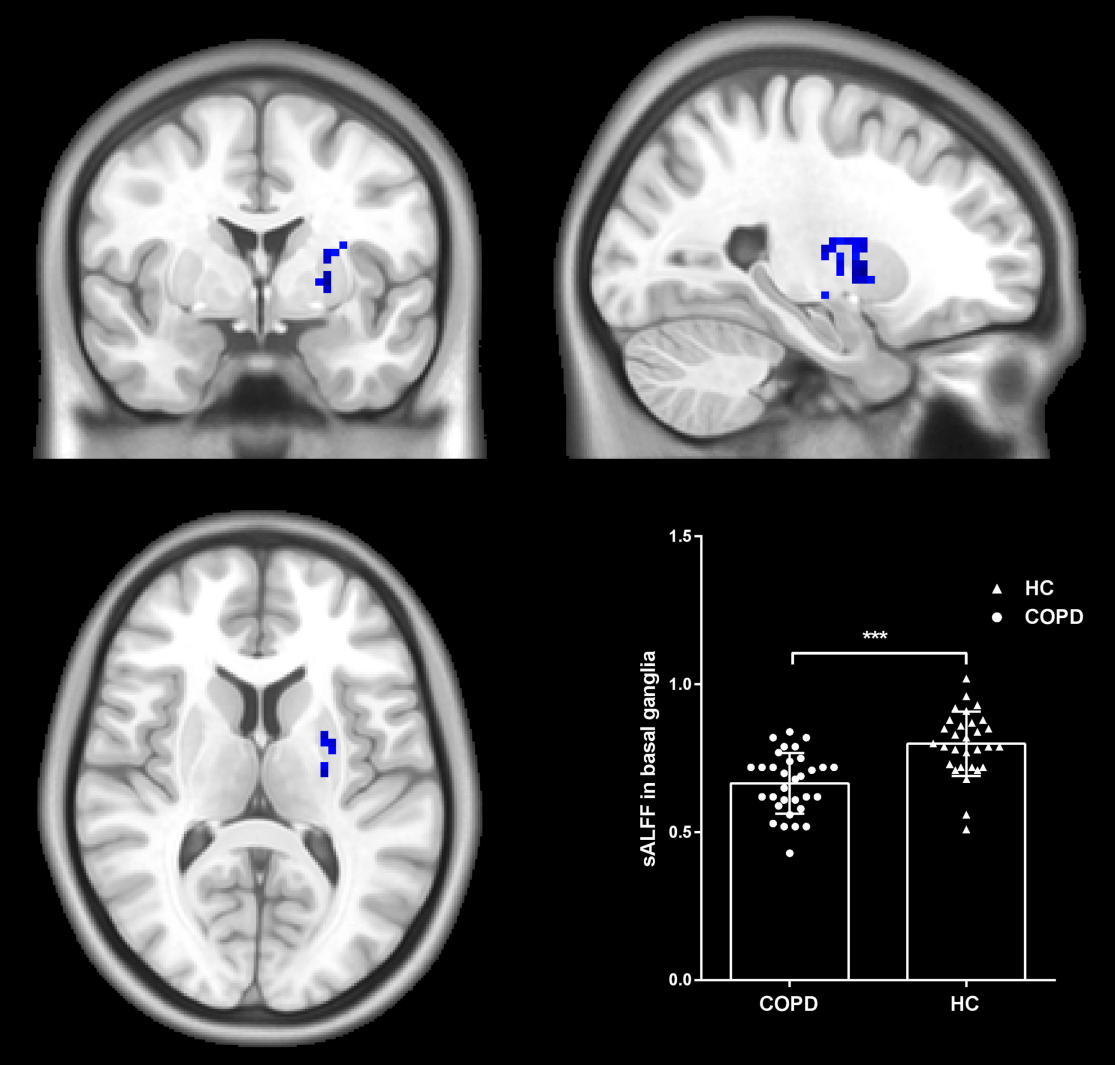
Group differences in sALFF. Patients with COPD showed decreased sALFF in the left basal ganglia compared to HC. Statistical maps were corrected via the Gaussian Random Field (GRF) method at a threshold of voxel *P* < 0.001, cluster *P* < 0.05 (with 7.6*8.0*6.7 mm^3^ as estimate smoothness kernel). Scatter plots present sALFF of each participant in the two groups. ***p < 0.001.

We explored the differences in sALFF between the two groups based on voxel level. Compared with HC, there were lower sALFF in the left basal ganglia (cluster size = 66; peak coordinate = −24, 0, 0) (shown in Figure 1). No other regions demonstrating significantly different sALFF between the two groups were found.

### Group Comparison of dALFF

**Figure 2:**
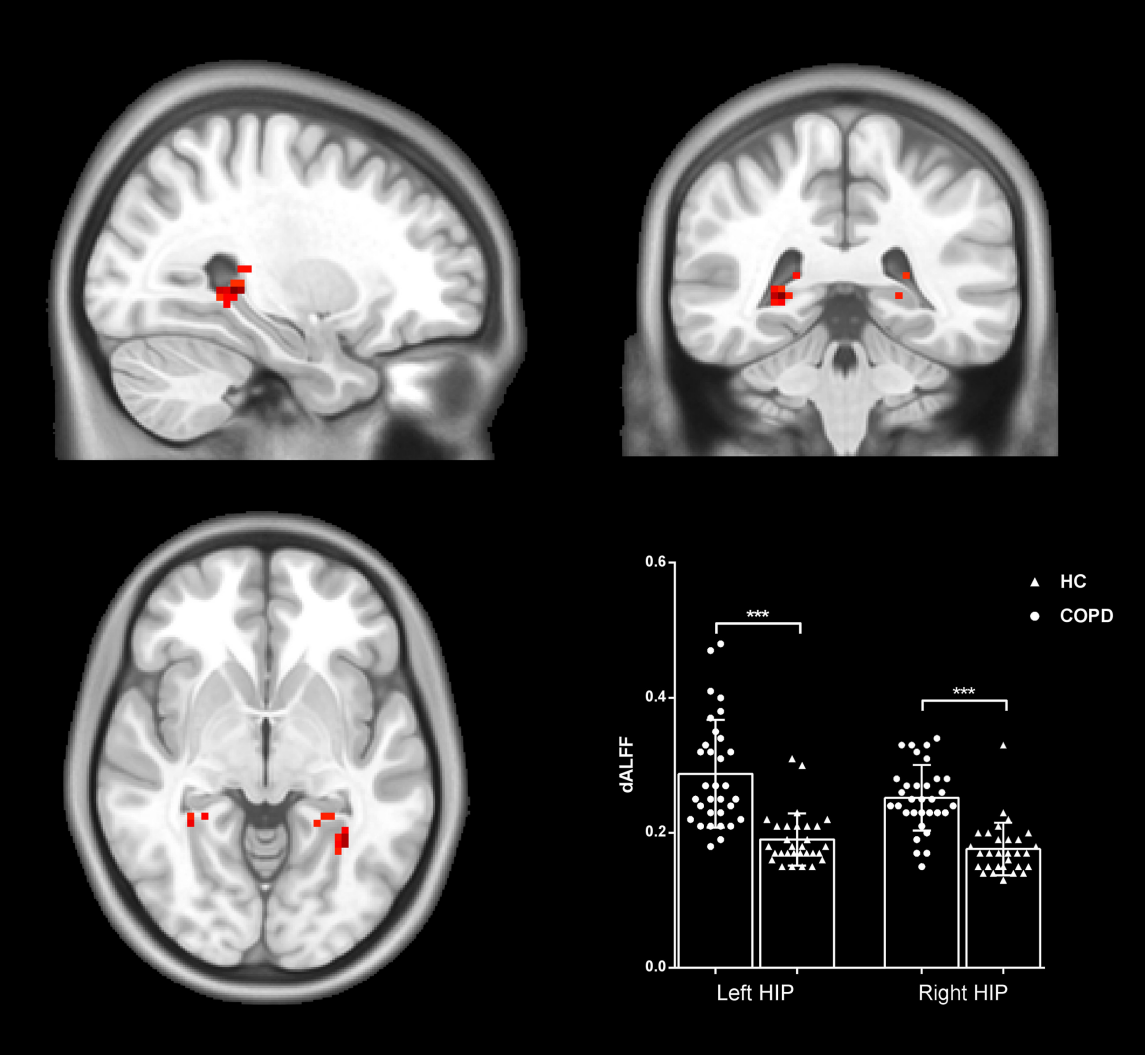
Group differences in dALFF. Patients with COPD showed increased dALFF in the bilateral hippocampal/parahippocampal cortex. Statistical maps were corrected via the Gaussian Random Field (GRF) method at a threshold of voxel *P* < 0.001, cluster *P* < 0.05 (with 9.5*10.2*8.7 mm^3^ as estimate smoothness kernel). Scatter plots present sALFF of each participant in the two groups. ***p < 0.001.

The differences in dALFF between the two groups were also examined based on voxel level. Compared with HC, patients with COPD exhibited decreased dALFF in the bilateral hippocampal/parahippocampal cortex (cluster = 48, peak coordinate = 27, −39, 0; cluster size = 50, peak coordinate = −27, −51, −3) (shown in Figure 2). The validation analysis with different sliding-window lengths also revealed decreased dALFF in the bilateral hippocampal/parahippocampal cortex in the COPD group (Supplementary Figure 1).

### Relationship Between sALFF and dALFF Clinical Characteristics and Behavioral Performance

The reduced sALFF in the left basal ganglia were lower oxygen partial pressure (*r* = 0.544, *p* = 0.001). Besides, the increased dALFF in the left hippocampal/parahippocampal cortex were associated with poor semantic-memory performance (*r* = 0.544, *p* = 0.001) and the increased dALFF in the left hippocampal/parahippocampal cortex were associated with the forced vital capacity (*r* = 0.544, *p* = 0.001). The sALFF in the left basal ganglia were negatively related with dALFF in the bilateral hippocampal/parahippocampal cortex (*r* = −0.360, *p* = 0.004 for the left and *r* = −0.341, *p* = 0.007). There was no significant relationship of other clinical characteristics with sALFF, or dALFF (see Table 2).

**Table 2:** Correlations between clinical characteristics and brain function.

	**PH**	**PaO_**2**_**	**PaCO_**2**_**	**FEV_**1**_**	**FVC**	**FEV_**1**_/FVC**	**CVFT**	**sALFF in Put**	**dALFF in LHIP**	**dALFF in RHIP**
PH	-	0.107	−0.514^**^	0.362^*^	0.196	0.441^*^	0.141	0.214	−0.014	−0.156
PaO_2_	-	-	−0.192	0.193	0.130	0.080	0.195	0.562^**^	0.089	0.219
PaCO_2_	-	-	-	−0.403^*^	−0.234	−0.437^*^	−0.157	−0.082	−0.148	0.216
FEV_1_	-	-	-	-	0.914^***^	0.478^**^	0.314	−0.080	−0.202	−0.300
FVC	-	-	-	-	-	0.140	0.265	−0.185	−0.231	−0.357^*^
FEV_1_/FVC	-	-	-	-	-	-	0.299	0.073	−0.146	−0.029
CVFT	-	-	-	-	-	-	-	0.030	−0.579^***^	−0.121
sALFF in BG	-	-	-	-	-	-	-	-	−0.360^**^	−0.341^**^
dALFF in LHIP	-	-	-	-	-	-	-	-	-	0.348
dALFF in RHIP	-	-	-	-	-	-		-	-	-

* *p < 0.05*;

** *p < 0.01*;

*** *p < 0.001*.

## Discussion

In the present study, we aimed to reveal brain functional alterations in COPD patients and their relationships to cognitive dysfunction. We found that COPD patients exhibited decreased local spontaneous activity in the left basal ganglia. We also observed a novel temporal dynamic alteration in the local spontaneous activity in the bilateral parahippocampal/hippocampal gyrus. Importantly, there were significant relationships between static and dynamic local spontaneous activities and clinical characteristics (both pulmonary function and semantic-memory performance).

Increasing evidence has suggested that patients with COPD display abnormal local spontaneous neuronal activity. Lu et al. have demonstrated lower local spontaneous activity in the bilateral basal ganglia among COPD patients indexed with static ALFF (sALFF), which is consistent with our results (Lu et al., 2019). Lower sALFF in the basal ganglia was associated with poor pulmonary function. Their deep location in the brain may contribute to the susceptibility of basal ganglia to persistent hypoxia (Schindler et al., 2016), due to neurovascular-coupling alterations. Basal ganglia are a group of subcortical nuclei that provide functions in the motor and multiple cognitive domains, including executive functions (Monchi et al., 2006). The reduced local activity has been linked with executive dysfunction in COPD patients and other neuropsychiatric disorders (Maciel et al., 2016; Lu et al., 2019). Notably, basal ganglia is also involved in semantic processing (Viñas-Guasch and Wu, 2017). Studies involving lesion imaging have suggested that lesions in the basal ganglia may impair verbal-fluency performance (Chouiter et al., 2016). Considering the fact that the verbal fluency test reflects both executive and semantic processing, we speculate that the lower local spontaneous activity in the basal ganglia may be related to executive dysfunction and semantic impairments.

Besides the basal ganglia, the hippocampal cortex is another brain structure that is sensitive to hypoxia (Dunn et al., 1999). This phenomenon may be attributed to the relative lack of capillary anastomoses between intrahippocampal vessels (Perosa et al., 2020). In line with this concept, our results revealed abnormal local activity in the hippocampal/parahippocampal cortex and the significant relationship between local activity and poor pulmonary function. The hippocampal/parahippocampal cortex is critical for episodic memory (Düzel et al., 2001) and consolidation of long-term memory (Frey and Frey, 2008) and contributes to many other cognitive domains, such as semantic memory (Sheldon and Moscovitch, 2012). Hippocampal dysfunction has been linked to semantic-memory impairments (Bai et al., 2019). Consistent with this, the present study has found an association between hippocampal activity and CVFT performance, which most likely relies on the medial temporal cortex, which mediates the storage and retrieval of semantic knowledge (Henry and Crawford, 2004).

Notably, this work took an innovative approach that investigated the temporal variability of local brain activity in COPD. Mounting studies suggest that brain dynamics reflect the functional capacity of the neural system (Kucyi et al., 2016) and more readily predict cognitive and affective conditions (Wang et al., 2019; Cui et al., 2020). Using a novel dALFF method, we revealed the abnormal enhanced dynamics of hippocampal local activity, which imply abnormal stability in the hippocampal local activity. Intriguingly, there were negatively significant relationships between sALFF in basal ganglia and dALFF in bilateral hippocampal/parahippocampal cortex. Physiological meanings of these relationships are still unclear. We speculate that the alteration of sALFF and dALFF may play an intermediary role between anoxia and memory impairments.

There are several limitations inherent in the present study. First, the sample size was too small, which may have led to sampling bias. Further studies with larger sample sizes are needed to replicate our results. Second, the data on arterial blood gas analysis and pulmonary function were lacking for healthy controls, although all healthy controls were screened rigorously by a physician to exclude possible hypoxemia and poor pulmonary function. Third, patients in the present study were not drug-free, and thus we cannot eliminate the confounding effects of drugs on our findings.

## Conclusion

These limitations notwithstanding, our results indicate that semantic-memory impairments in COPD patients are linked with abnormal static and dynamic local-neural activity in the basal ganglia and parahippocampal/hippocampal cortex, which may be modulated by poor pulmonary function.

## Data Availability Statement

The raw data supporting the conclusions of this article will be made available by the authors, without undue reservation.

## Ethics Statement

The studies involving human participants were reviewed and approved by the Hefei Second People's Hospital Ethics Committee. The patients/participants provided their written informed consent to participate in this study.

## Author Contributions

ZL, QC, YW, and XF designed the study. YJ, PH, and LZ acquired behavior and imaging data. TB and KW help for analyzing the clinical and imaging data. ZL, QC, and XF wrote this article, which all authors have reviewed. All authors contributed to the article and approved the submitted version.

## Conflict of Interest

The authors declare that the research was conducted in the absence of any commercial or financial relationships that could be construed as a potential conflict of interest.
