# Supplementary figures and images for: Abnormal Static and Dynamic Local-Neural Activity in COPD and Its Relationship With Pulmonary Function and Cognitive Impairments
Source: Front Hum Neurosci. 2021 Jan 15;14:580238. doi: 10.3389/fnhum.2020.580238 (PMC7843446; doi:10.3389/fnhum.2020.580238)

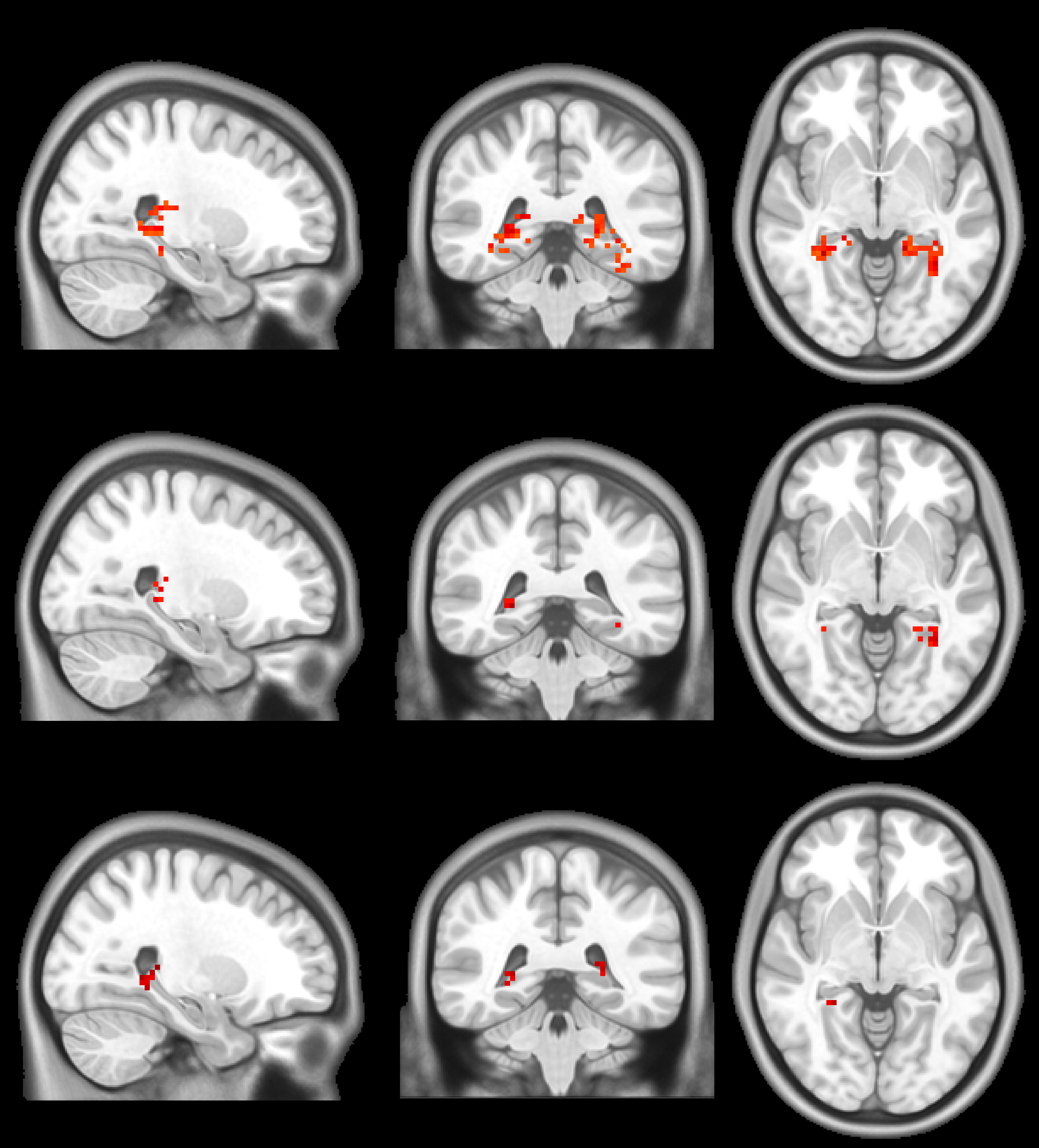

Supplement: Supplementary file 1 [file Image_1.TIF]
